# Modulation of MUC1 mucin as an escape mechanism of breast cancer cells from autologous cytotoxic T-lymphocytes

**DOI:** 10.1054/bjoc.2000.1744

**Published:** 2001-05

**Authors:** K Kontani, O Taguchi, T Narita, M Izawa, N Hiraiwa, K Zenita, T Takeuchi, H Murai, S Miura, R Kannagi

**Affiliations:** Program of Molecular Pathology andDepartment of Breast Surgery, Aichi Cancer Center, 11 Kanokoden, Chikusa-ku, Nagoya 4648681, Japan

**Keywords:** immunotherapy of cancer, breast cancer, mucin, carbohydrate determinants

## Abstract

MUC1 mucin is known to serve as a target molecule in the killing of breast cancer cells by cytotoxic T-lymphocytes (CTLs). We searched for a possible mechanism allowing tumour cells to escape from autologous CTLs. When the killing of breast cancer cells by autologous lymphocytes was examined in 26 patients with breast cancer, significant tumour cell lysis was observed in 8 patients, whereas virtually no autologous tumour cell lysis was detected in as many as 18 patients. In the patients who showed negligible tumour cell lysis, the autologous tumour cells expressed MUC1-related antigenic epitopes much more weakly than the tumour cells in the patients who exhibited strong cytotoxicity (significant statistically at *P*< 0.0005–0.0045), suggesting that the unresponsiveness of cancer cells to CTLs observed in these patients was mainly due to loss of MUC1 expression or modulation of its antigenicity. A breast cancer cell line, NZK-1, established from one of the cytotoxicity-negative patients, did not express MUC1 and was resistant to killing by CTLs, while control breast cancer cell lines expressing MUC-1 were readily killed by CTLs. Transfection of NZK-1 cells with MUC1 cDNA induced significant lysis by autologous T-lymphocytes. These results supported the importance of MUC1 mucin in autologous anti-tumour immunity, but suggested that the major escape mechanism of tumour cells from autologous T-lymphocytes is the loss and/or modulation of MUC1 antigenicity on tumour cells, which would limit the effectiveness of possible immunotherapy designed to target the MUC1 mucin. © 2001 Cancer Research Campaign http://www.bjcancer.com


*British Journal of Cancer* (2001) **84**(9), 1258–1264
© 2001 Cancer Research Campaign
doi: 10.1054/ bjoc.2001.1744, available online at http://www.idealibrary.com on IDEAL®

### Modulation of MUC1 mucin as an escape mechanism of breast cancer cells from autologous cytotoxic T-lymphocytes

**K Kontani[1], O Taguchi[1], T Narita[1], M Izawa[1], N Hiraiwa[1], K Zenita[1], T Takeuchi[2], H Murai[2], S Miura[2] and R Kannagi[1]**

[1]Program of Molecular Pathology and [2]Department of Breast Surgery, Aichi Cancer Center, 1–1 Kanokoden, Chikusa-ku, Nagoya 464–8681, Japan

**Summary** MUC1 mucin is known to serve as a target molecule in the killing of breast cancer cells by cytotoxic T-lymphocytes (CTLs). We searched for a possible mechanism allowing tumour cells to escape from autologous CTLs. When the killing of breast cancer cells by autologous lymphocytes was examined in 26 patients with breast cancer, significant tumour cell lysis was observed in 8 patients, whereas virtually no autologous tumour cell lysis was detected in as many as 18 patients. In the patients who showed negligible tumour cell lysis, the autologous tumour cells expressed MUC1-related antigenic epitopes much more weakly than the tumour cells in the patients who exhibited strong cytotoxicity (significant statistically at $P < 0.0005$–$0.0045$), suggesting that the unresponsiveness of cancer cells to CTLs observed in these patients was mainly due to loss of MUC1 expression or modulation of its antigenicity. A breast cancer cell line, NZK-1, established from one of the cytotoxicity-negative patients, did not express MUC1 and was resistant to killing by CTLs, while control breast cancer cell lines expressing MUC-1 were readily killed by CTLs. Transfection of NZK-1 cells with MUC1 cDNA induced significant lysis by autologous T-lymphocytes. These results supported the importance of MUC1 mucin in autologous anti-tumour immunity, but suggested that the major escape mechanism of tumour cells from autologous T-lymphocytes is the loss and/or modulation of MUC1 antigenicity on tumour cells, which would limit the effectiveness of possible immunotherapy designed to target the MUC1 mucin. © 2001 Cancer Research Campaign http://www.bjcancer.com

**Keywords**: immunotherapy of cancer; breast cancer; mucin; carbohydrate determinants

The human epithelial mucin MUC1 is a high molecular glycoprotein characterized by heavy glycosylation, and is known to be expressed on various human cancers including mammary, pancreatic gastric and colon cancers (Burchell et al, 1987; Lan et al, 1990; Ogata et al, 1992; Ho et al, 1995; Hakomori, 1996; Taylor-Papadimitriou and Finn, 1997). What distinguishes MUC1 from other tumour-associated antigens is that MUC1 mucin was demonstrated to be strongly recognized by CTLs in a MHC- unrestricted manner (Barnd et al, 1989). The mechanism of the MUC1 recognition by T-lymphocytes has extensively been studied (Barnd et al, 1989; Agrawal et al, 1996; Pecher and Finn, 1996; Magarian-Blander et al, 1998).

Approximately 20 to 100 tandem repeats of the conserved 20 amino acid sequences in the MUC1 core protein are thought to assure adequate affinity with antigen-binding regions of TCR (Swallow et al, 1987; Gendler et al, 1988, 1990; Siddiqui et al, 1988), and this is proposed to activate cytotoxic T-lymphocytes (CTLs), even if the antigenic peptide is not presented in the context of MHC molecules. The exposure of the epitopes in the tandem repeat domain was also known to be significantly affected by carbohydrate structures bound on the core protein (Bara et al, 1993). Normal epithelial cells express MUC1, but its glycosylation on normal epithelial cells was reported to be different from that on cancer cells (Hanisch et al, 1989; Lloyd et al, 1996). Long and complexly branched carbohydrates on normal epithelial cells ordinarily mask MUC1 core proteins at the cell surface, while MUC1 in cancer cells express much shorter carbohydrate chains, such as sialylated or non-sialylated Tn- and TF-antigens, and this aberrant glycosylation of MUC1 is proposed to be important in inducing significant CTL response in patients with cancer. Even more direct involvement of certain carbohydrate determinants in T-cell recognition is suggested (Böhm et al, 1997).

MUC1 is assumed to be a good candidate for adoptive immunotherapy against human cancers (Akagi et al, 1997; Karanikas et al, 1997; Samuel et al, 1998; Acres et al, 2000; Carmon et al, 2000; Wright et al, 2000; Kontani et al, 2000). However, the reaction of autologous CTLs against MUC1 mucin expressed on autologous tumour cells in patients has not been thoroughly characterized. For the development of effective immunotherapy against cancer, it would be important to use the autologous experimental system. In this study, we investigated the possible relationship between expression of MUC1 mucin on cancer cells and the lytic activity of autologous CTLs, and attempted to clarify the mechanism by which autologous cancer cells escape from the lytic activity of autologous lymphocytes.

### MATERIALS AND METHODS

### Cell lines, monoclonal antibodies and other reagents

Cultured human cell lines, ZR-75-1, BT-20, and T-47D were obtained from American Type Culture Collection (Rockville, MD). Monoclonal antibodies, DF3, 115D8, B72.3 and CC49 were provided from Toray-Fuji Bionics Inc. (Tokyo, Japan). SM3 was purchased from Cymbus Bioscience Ltd. (Southampton, UK), and TKH6 was from Otsuka Pharmaceutical Co (Tokushima, Japan). The speficities of these antibodies are summarized in Table 1. SNH3, which is reactive to sialyl-Le$^X$ (Takada et al, 1993), was a

*Received 17 August 2000*
*Revised 24 January 2001*
*Accepted 25 January 2001*

*Correspondence to:* R Kannagi

**Table 1** Specificity of antibodies used for detection of antigenic determinants carried by MUC-1 mucin

| Antibodies | Antigenic structures detected |
|---|---|
| Anti-MUC-1 | |
| DF3 | TRPAPGS |
| SM3 | PDTRP |
| 115D8 | Glycopeptide[a] |
| Anti-carbohydrate | |
| TKH6 | GalNAcα-*O*-Ser/Thr |
| B72.3 | NeuAcα 2→ 6GalNAcα1-*O* -Ser/Thr(Sialyl Tn) |
| CC49 | NeuAcα2 → 6(Galβ1→3) GalNAcα 1-*O* -Ser/Thr |
| SNH3[b] | NeuAcα2 →3Galβ1→4 (Fucα1 →3)GlcNAc β1→R |

GalNAc, *N*-acetyl-galactosamine; NeuAc, *N*-acetyl-neuraminic acid; Gal, galactose; Fuc, fucose; GlcNAc, *N*-acetyl-glucosamine. [a]115D8 is reactive to fully glycosylated MUC1 as well as underglycosylated tumour-associated MUC1. However, it failed to react to any of the MUC1 peptides of various length so far tested in the Tumor Differentiation-4 workshop (Blockzjil et al, 1998; Price et al, 1998). The antibody also failed to react to any hitherto-known carbohydrate determinants, while a periodate-treatment inhibited the binding (Dai et al, 1998). [b]SNH3 was used as a control anti-carbohydrate antibody since the determinant defined by this antibody (sialyl $Le^x$) is known to be carried frequently by MUC-1 mucin, but is not thought to be intimately involved in cancer cell lysis by CTLs.

kind gift from Dr Sen-itiroh Hakomori, Biomembrane Institute, Seattle, WA. Anti-human CD4 and CD8 monoclonal antibodies were purchased from Seikagaku Corp (Tokyo, Japan), and anti-human TCRαβ monoclonal antibody was from Cosmo Bio Co, Ltd (Tokyo, Japan). Dispase was purchased from Godo Shusei Co Ltd (Tokyo, Japan), Ficoll-Conray Lymphosepal from Immuno-Biological Laboratories (Gunma, Japan) and $Na_2{}^{51}CrO_4$ from DuPont-NEN (Boston, MA). Sialidase and Geneticin (G418) were from GIBCO BRL (Tokyo, Japan).

### Flow cytometric analysis

Breast cancer cell lines were suspended in PBS and incubated with anti-MUC1 monoclonal antibodies diluted on ice for 30 min. After 3 washes in cold PBS, the cells were incubated with fluorescein isothiocyanate-conjugated anti-mouse Ig diluted at 1/1000 on ice for 30 min. Flow cytometric analyses were performed using a FACScan flow cytometer (Becton Dickinson, San Jose, CA) following 3 washes in cold PBS.

### Tumours from patients with breast cancer and immunohistochemical study

26 female patients ranging in age from 32 to 77 and in clinical stage from 1 to 3 were studied. Clinical features of the patients are summarized in Table 2. A part of tumour specimens obtained from the main breast tumours from these patients was either fixed in 10% formalin and embedded in paraffin or frozen in OCT compound in liquid nitrogen. Paraffin-embedded sections were incubated with monoclonal antibodies diluted to appropriate concentrations in PBS at room temperature for 2 h following pre-incubation in 0.2% hydrogen peroxide-containing methanol to block non-specific binding sites. After 3 washes in PBS, the sections were incubated with peroxidase-conjugated anti-mouse IgG diluted at 1/200 in PBS at room temperature for 30 min. They were washed in PBS 3 times and incubated in 5 mM Tris-HCl containing diaminobenzidine to visualize for 10 min. After washing in distilled water, nuclear compartments of cancer cells were stained in haematoxylin solution. After dehydration in 100% ethanol, the sections were mounted in 50% glycerin. Frozen sections were fixed in 50% chloroform solution and stained according to the indirect peroxidase-staining technique described above. The percentage of positive cells was calculated by the number of cancer cells positively stained out of 500 cancer cells counted in each specimen.

### Preparation of breast cancer cells from patients with breast cancer

Breast cancer cells were freshly isolated from main breast tumours of the above patients at the time of surgical operations. The study was performed with approval by the institutional review board. Resected tumour samples were minced and incubated in RPMI 1640 supplemented with Dispase at the concentration of 10 000 PU $ml^{-1}$ at 37˚C for 60 min to obtain single cell suspensions. The suspensions were layered on the top of step gradients, from the bottom comprising 100% and 75% Lymphosepal and centrifuged at 400 × ***g*** for 30 min. Tumour cells were collected from the interface of the sample and 75% Lymphosepal layer and resuspended in RPMI 1640 containing 2% FCS. A MUC1-deficient cell line, NZK-K1, was established from a main tumour of a 45-year-old female breast cancer patient, by grafting the tumour specimens subcutaneously in female SCID mice, which were purchased from Charles River Inc (Hino, Japan). NZK-K1 cells completely lacked expression of MUC1 mucin at the cell surface as well as expression of MUC1 mRNA as ascertained by Northern blotting.

**Table 2** Clinical features of breast cancer patients studied

| Patient no. | Age | Clinical stage | Histological type |
|---|---|---|---|
| 1 | 45 | t2n1m0 | SC[a] |
| 2 | 61 | t2n0m0 | PT[b] |
| 3 | 39 | t1n0m0 | PT |
| 4 | 64 | t2n0m0 | SC |
| 5 | 50 | t2n0m0 | SC |
| 6 | 77 | t2n0m0 | SC |
| 7 | 68 | t3n0m0 | SC |
| 8 | 61 | t3n0m0 | SC |
| 9 | 67 | t3n1m0 | Med.[c] |
| 10 | 47 | t2n0m0 | PT |
| 11 | 59 | t2n1m0 | Med. |
| 12 | 53 | t2n0m0 | ST[d] |
| 13 | 42 | t4n0m0 | SC |
| 14 | 54 | t2n0m0 | ST |
| 15 | 45 | t2n0m0 | SC |
| 16 | 45 | t2n0m0 | SC |
| 17 | 71 | t2n0m0 | PT |
| 18 | 35 | t2n0m0 | SC |
| 19 | 60 | t2n1m0 | SC |
| 20 | 67 | t3n0m0 | SC |
| 21 | 46 | t2n0m0 | SC |
| 22 | 44 | t1n0m0 | Med. |
| 23 | 38 | t3n2m0 | Med. |
| 24 | 47 | t2n0m0 | PT |
| 25 | 32 | t2n0m0 | ST |
| 26 | 56 | t2n0m0 | Comedo |

[a] Scirrhous type; [b] Papillo-tubular type; [c] Medullary type; [d] Solid tubular type.

© 2001 Cancer Research Campaign

### Preparation of peripheral blood mononuclear cells (PBMC) and cytotoxicity assay

Heparinized peripheral blood samples obtained from the patients were diluted in the same volume of PBS, layered on Lymphosepal and centrifuged at 400 × ***g*** at room temperature for 30 min. PBMCs were collected from the interface of the sample and the Lymphosepal layer, then resuspended in RPMI 1640 containing 2% FCS.

Breast cancer cell lines or cancer cells obtained from cancer patients were incubated with $Na_2{}^{51}CrO_4$ at 37˚C for 60 min. Radio-labelled $10^4$ cells were transferred to a 96-well U-bottom culture plate containing $5 \times 10^5$ PBMCs prepared in 200 µl RPMI 1640 supplemented with 10% FCS and $5 \times 10^{-5}$ M 2-mercaptoethanol following 3 washes in culture medium. They were incubated at 37˚C for 18 hours to measure the cytotoxicity as previously reported (Finke et al, 1990; Smyth and Ortaldo, 1993; Kontani et al, 2000). For inhibiting the recognition of antigenic structures by effector cells, target cells were pre-incubated with anti-MUC1 monoclonal antibodies at 37˚C for 45 min prior to co-culture with effector cells. For characterization of effector cell population responsible for the tumour cell lysis, PBMCs were treated with anti-TCRαβ, -CD4 or -CD8 antibodies at 37˚C for 45 min followed by co-incubation with radio-labelled target cells. After incubation of target cells and PBMCs for 18 h, the supernatant was collected from each well and counted for the release of $^{51}Cr$ from the lysed target cells, using LKB-Wallac Minigamma 1275 (Wallac Oy, Finland). The percentage of chromium release was determined by the formula: % Lysis = 100 × (Experimental release–Spontaneous release)/ (Maximal release–Spontaneous release).

### Transfection of a breast cancer cell line with MUC1 cDNA

MUC1 cDNA expression vector, pCMV5-PEM (Gendler et al, 1990), was a kind gift from Dr Sandra J Gendler (Mayo Clinic Scottsdale, Scottsdale, Arizona). Transfection of a MUC1-deficient cell line, NZK-K1, with the cDNA was performed using the electroporation technique as described (Potter et al, 1984). Briefly, NZK-K1 cells, 400 µl of $5 \times 10^6$ cells $ml^{-1}$ in Dulbecco's PBS buffer, were transfected with 1 µg of pDKOF.muc1 using a ECM600® Electro-Cell Manipulator (BTX Inc, San Diego, USA) as follows: 400 µl of $5 \times 10^6$ cells $ml^{-1}$ in Dulbecco's PBS buffer were pulsed at 150 V, 72 ohms, 500 µGarads in a sterile electroporation cell. Aliquots of the electroporated cells were added to normal medium and grown for 2 days. G418 was added at 600 µg $ml^{-1}$ to select stable transfectants. Resistant clones appeared within 4–5 weeks, and colonies of independent transfectants were obtained by limiting dilution and screened for expression of the MUC1 epitopes.

### RESULTS

### Cytotoxic activity of PBMCs from patients with breast cancer against autologous cancer cells and its correlation to MUC1 expression on cancer tissues

When freshly isolated PBMCs from the patients were co- incubated with autologous cancer cells, 8 (31%) out of 26 cases showed statistically significant positive cytotoxic activity for autologous cancer cells (Figure 1). As many as 18 patients, however, displayed only negligible killing activity. This did not seem to be due to the malfunction of lymphocytes prepared from the patients, since significant cytolytic activity was observed when well-known cultured breast cancer cell lines were used as target cells instead of autologous cancer cells in some assays. During the course of this study, we noticed that the degree of killing correlated well with the expression of MUC1-related determinants on autologous cancer cells.

Accordingly, the patients were divided into 2 groups of cytotoxicity-positive ($n$ = 8) and -negative ($n$ = 18) cases, respectively. The immunohistochemical expression of MUC1-tandem repeat epitopes and related carbohydrate determinants was carefully examined and compared between the 2 groups. The results indicated that the expression of MUC1 tandem repeat epitope defined by the DF3 antibody and that of glycopeptide epitope defined by the 115D8 antibody were significantly higher in the cytotoxicity-positive cases than in the cytotoxicity-negative cases (Figure 2). Among MUC1-related carbohydrate determinants, expression of the CC49-defined determinant turned out to be significantly higher in the cytotoxicity-positive cases compared to that in the cytotoxicity-negative cases (Figure 2). It was noted that the calculated statistical significance was as high as $P$ = 0.0005 for the DF3 antibody, 0.0045 for CC49, followed by 0.0083 for 115D8.

All of the 8 patients with positive cytotoxicity showed strong MUC1-core epitope expression as well as CC49-defined carbohydrate determinants in their tumours. On the other hand, among the 18 cytotoxicity-negative cases, 14 showed markedly reduced or no expression of the MUC1 epitopes and/or CC49-related carbohydrate determinants. These results suggested that the major cause of unresponsiveness of CTLs in cytotoxicity-negative cases to autologous tumour cells was due to the loss or decreased expression of MUC1-related epitopes, rather than any functional disturbance of lymphocytes. There were only 4 cases in which tumour cells expressed MUC1 epitopes and CC49-defined determinants at a level comparable to those in cytotoxicity- positive cases, nevertheless being unresponsive to autologous CTLs.

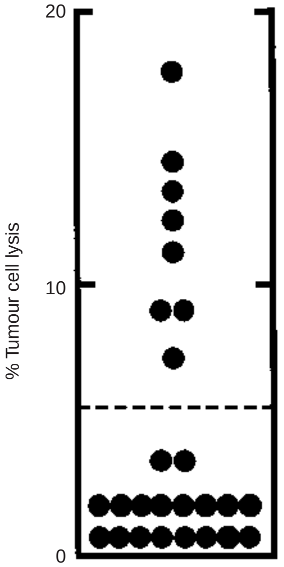

**Figure 1** Cytotoxic activity of PBMCs from patients with breast cancer against autologous cancer cells. Breast cancer cells, prepared as described in 'Material and Methods', were $^{51}Cr$-labelled and incubated with freshly isolated PBMCs for 18 h. The percentage of tumour cell lysis was plotted for each patient. Lytic activity more than 6.3% was statistically significant ($P$ < 0.05)

 *© 2001 Cancer Research Campaign*

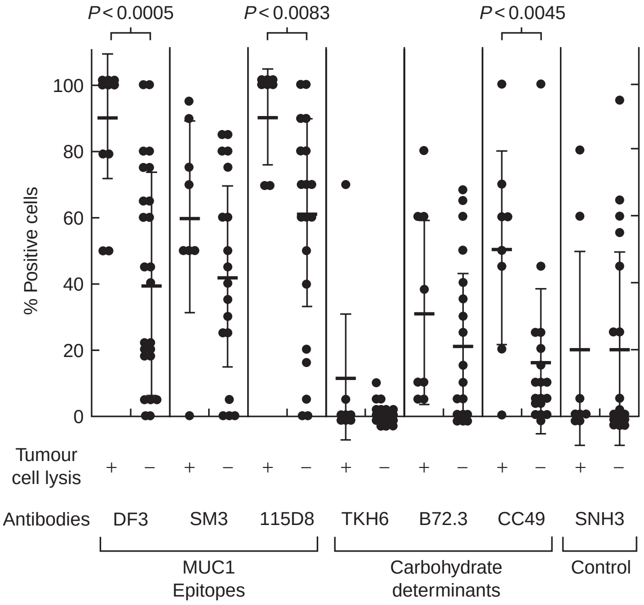

**Figure 2** Relationship between MUC1 expression in cancer cells and its ability to elicit anti-tumour immunity. The patients studied were divided into 2 groups, cytotoxicity-positive ($n = 8$) and -negative ($n = 18$) cases, according to the result of the autologous cytotoxicity assay. Mean percentages of cancer cells positive for the reactivity to each anti-MUC1 antibody were compared between the 2 groups. Statistical analyses were performed by Student's *t*-test

### Isolation of cytotoxicity-resistant breast cancer cell line from non-responder patients

We next established a breast cancer cell line, NZK-K1, from cancer tissues of one of the patients showing negligible autologous cytotoxic activity. This cell line completely lacked the expression of MUC1 epitopes defined by all 6 monoclonal antibodies in flow cytometric analysis (Figure 3, upper panel). The cells also lacked the MUC1 mRNA expression ascertained by Northern blotting, while expressing a high level of class I MHC molecules. The NZK-K1 cells were not lysed by PBMCs prepared from autologous peripheral blood or from healthy donors, while the control cultured breast cancer cell lines such as ZR-75–1, BT-20 and T-47D were significantly killed by the same PBMCs as shown in Figure 4A.

The killing of the ZR-75–1, BT-20 and T-47D cells by PBMCs was inhibited in varying degrees by the DF3, 115D8 or SM3 antibodies directed to the MUC1 epitopes (Figure 4B). These 3 cell lines significantly expressed the epitopes defined by SM3 as well as DF3 or 115D8 on flow cytometric analyses (data not shown). The killing was also inhibited by the addition of the CC49 and to a lesser extent by the B72.3 antibodies, which define the MUC1-related carbohydrate determinants (Figure 4B). The killing of these control cell lines by PBMCs was virtually not inhibited by antibodies against class I or II MHC antigens (Figure 4B). Anti-TCRαβ as well as CD4 antibodies were shown to inhibit the lysis of target cells by PBMCs, while anti-CD8 antibody did not affect the cytotoxicity (Figure 4C). This suggested that the CTLs participating in the killing of these cells are MHC-non-restricted, and are $CD4^+$ $TCR\alpha\beta^+$ lymphocytes as characterized earlier (Barnd et al, 1989; Jerome et al, 1993).

### Induction of cytotoxic activity against MUC1 cDNA-transfected autologous breast cancer cell line

We transfected MUC1 cDNA to the NZK-K1 cells, and obtained a subclone, termed NZK-muc. The NZK-muc cells moderately expressed the MUC1 epitopes defined by the DF3 and 115D8 antibodies (Figure 3, middle panel). The SM3-defined epitope and MUC1-related carbohydrate determinants were, however, only weakly expressed on the NZK-muc cells (Figure 3, middle panel). Sialidase treatment of the transfectant cells enhanced the expression levels of the SM3 tandem repeat epitope and of

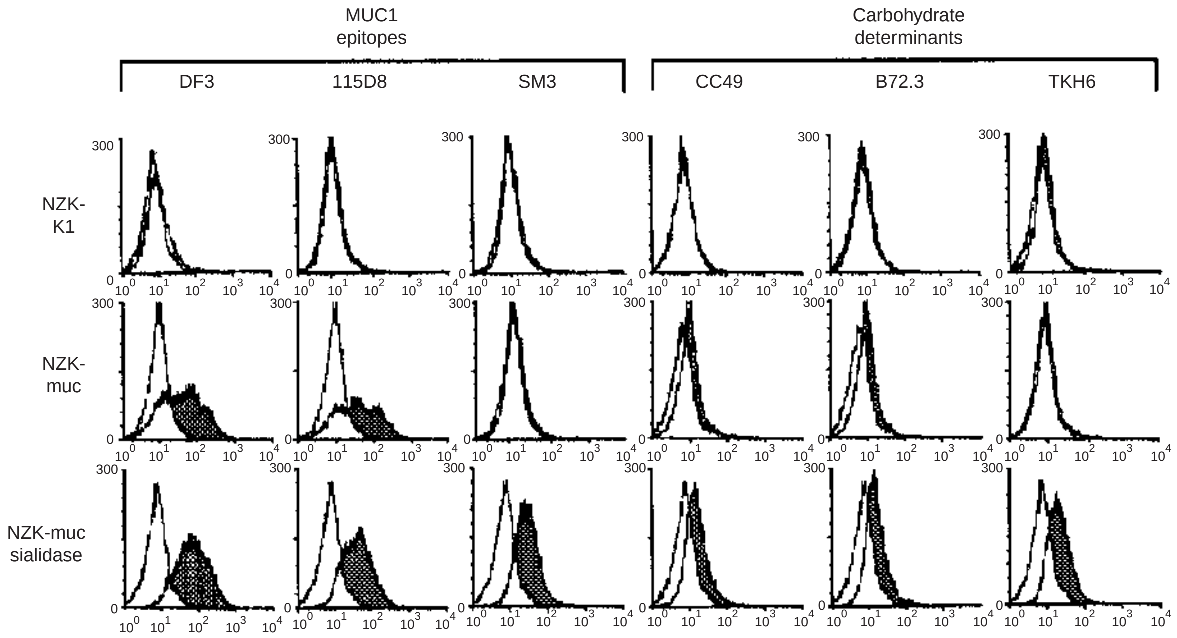

**Figure 3** Phenotypic analysis of MUC1 transfectants by flow cytometry. The MUC1 deficient parent cell line, NZK-K1, a MUC1 transfectant, NZK-muc, and sialidase-treated NZK-muc were stained with anti-MUC1 antibodies. MUC1 expression of these cell lines was studied as described in 'Materials and Methods'

© 2001 Cancer Research Campaign 

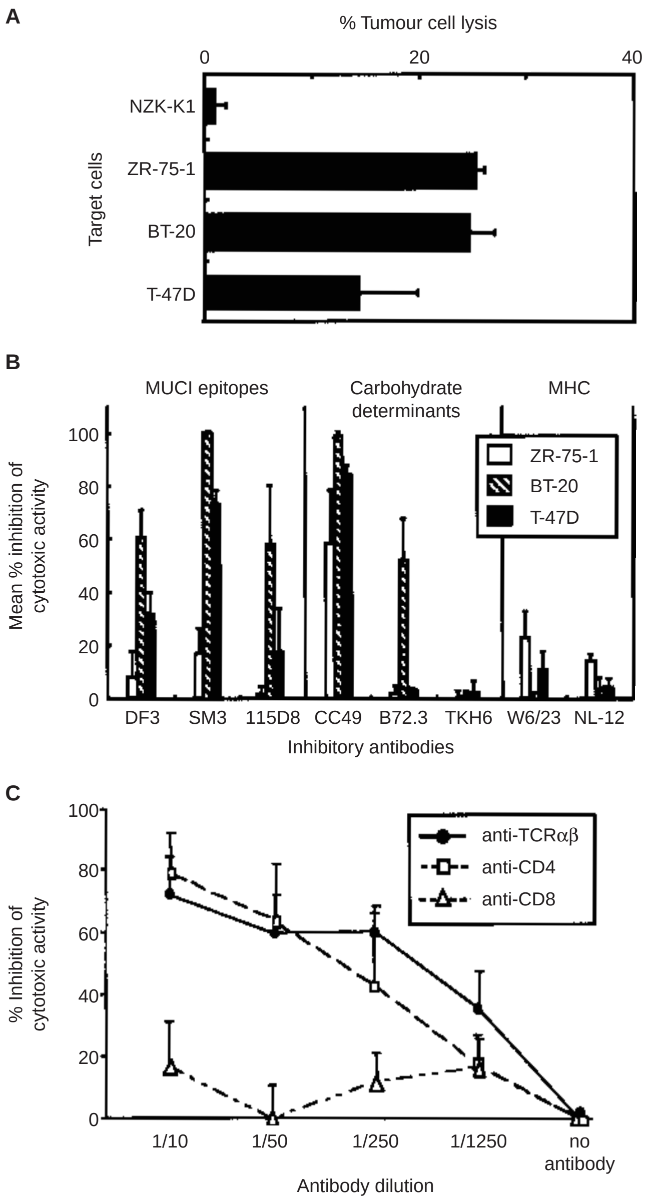

**Figure 4** Cytotoxic activity of PBMCs against cultured breast cancer cell lines. In panel **A**, $^{51}$Cr-labelled $1 \times 10^4$ breast cancer cells were incubated with $5 \times 10^5$ PBMCs obtained from normal donors in each well for 18 h as indicated in 'Materials and Methods'. NZK-K1 is the cell line established from non-responder. Cultured breast cancer cell lines ZR-75-1, BT-20 and T-47D serve as positive control. The effector/target ratio was 50:1. Panel **B**, inhibitory effect of anti-MUC1 monoclonal antibodies on the cytotoxicity against breast cancer cell lines. ZR-75-1, BT-20 and T-47D, were pretreated with anti-MUC1 antibodies or anti-MHC antibodies at 37˚C for 45 min prior to co-incubation with PBMCs. In **C** PBMCs were pretreated with anti-TCR α β, -CD4 or -CD8 antibodies at 37˚C for 45 min prior to co-cultivation with radio-labelled target cells, ZR-75-1

mucin-associated carbohydrate determinants, suggesting some sialylated carbohydrate side chains had masked expression of the mucin associated epitopes on untreated NZK-muc transfectant cells (Figure 3, lower panel).

When NZK-K1 or NZK-muc cells were used as target cells in cytotoxic assays with autologous PBMCs prepared from the same patient, the parent NZK-K1 cells were scarcely killed by the PBMCs, while significant killing activity was observed with NZK-muc transfectant (Figure 5). When sialidase-treated

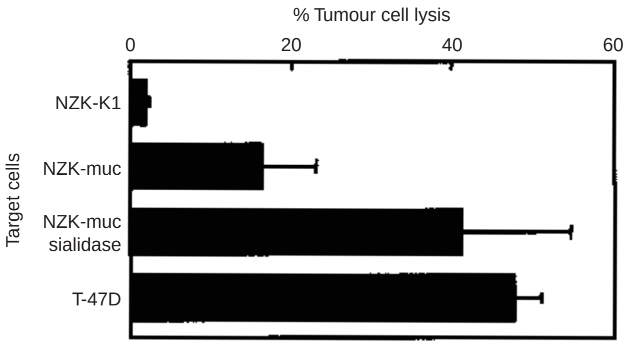

**Figure 5** Cytotoxic activity of autologous PBMCs against MUC1 transfectants. $^{51}$Cr-labelled NZK-K1, NZK-muc, and sialidase-treated NZK-muc cells were incubated with autologous PBMCs at effector/target ratio of 50:1 for 18 hours. The cultured breast cancer cell line T-47D served as a positive control for the cytotoxic activity. The amounts of isotope released from target cells were calculated as described in 'Materials and Methods'

NZK-muc cells were used as target cells, the autologous PBMCs exhibited strong lytic activity comparable to that obtained with the high MUC1 expressor T-47D, which served as a positive control in these assays (Figure 5). This indicated that autologous CTLs in the peripheral blood contained significant killing activity against tumour cells, and that the unresponsiveness of autologous CTLs initially observed with this patient was due to the loss of MUC1 antigenic epitopes from the surface of autologous cancer cells.

### DISCUSSION

In this study we investigated cytotoxicity of autologous lymphocytes against autologous tumour cells in patients with breast cancer. We confirmed an earlier notion (Barnd et al, 1989; Magarian-Blander et al, 1996, 1998) that certain T-lymphocyte populations that recognize the MUC1 mucin occur in peripheral blood of patients with breast cancers, as well as normal individuals, without any priming in vitro. Effector cells involved in the MUC1-mediated killing reaction were mainly $TCR\alpha\beta^+CD4^+$ T-lymphocytes, and the recognition of the MUC1 mucin was largely MHC-independent. It would seem unlikely that T-lymphocytes in freshly isolated PBMCs from normal donors could recognize the tumour antigen without any prior in vitro priming. However, naive T-lymphocytes may have a chance to encounter mucin shed from inflamed glandular epithelial cells, or processed and presented by phagocytes following apoptotic death of the epithelial cells even in normal individuals. Levels of serum soluble MUC1 mucin are known to be elevated in patients with cancer, and even the basal levels of the mucin are detected in the sera of normal individuals as well (Burchell et al, 1984; Devine et al, 1993; Willsher et al, 1993). The presence of MUC1-reactive T-lymphocytes is reported to occur in multiparous women (Agrawal et al, 1996), and even humoral antibodies directed to MUC1 mucin appear in the sera of cancer patients (Kotera et al, 1994; von Mensdorff-Pouilly et al, 2000). These findings suggest that some populations of T-lymphocytes have already been primed with MUC1 mucin and can be re-activated immediately in response to cancer cells expressing the mucin. It is possible that cancer patients as well as some normal individuals may have already in vivo-primed T-lymphocytes specific for MUC1 mucin in their PBMCs (Jerome et al, 1995, 1997).

© 2001 Cancer Research Campaign

A significant correlation was observed between the expression of the MUC1 epitopes in cancer tissues and the killing activity of autologous lymphocytes against the cancer cells in patients with breast cancer. These antibodies directed to MUC1 tandem repeats were effective in inhibiting the lysis of the target cells in cytotoxicity assay employing well-known breast cancer cell lines. Some antibodies directed to the mucin-associated aberrant carbohydrate determinants, such as CC49, also exerted a comparable inhibitory effect on the lysis of tumour cells. Expression of CC49-defined carbohydrate determinants in autologous cancer cells was also found to significantly correlate with killing activity of CTLs in patients with breast cancer. Normal glycosylation of MUC1 is known to inhibit the recognition of the molecule by CTLs, and effective induction of lytic activity is proposed to be achieved when the MUC1 molecule is aberrantly glycosylated and the antigenic epitopes are exposed on the surface (Kotera et al, 1994; Jerome et al, 1997). In line with this, all of the cancer patients with significant lytic activity in the autologous cytotoxicity assays demonstrated a strong expression of MUC1 epitopes as well as MUC1-related aberrant carbohydrate determinants on the cancer cells. These results collectively indicate that the susceptibility of the cancer cells to MUC1-specific CTLs is highly dependent on the appropriate expression of the antigenic epitopes on cancer cells.

T-cell tolerance towards tumour cells is recently suggested to be a major hindrance to the development of effective immune responses against tumours from experiments using human MUC1 transgenic mice (Gong et al, 1998; Rowse et al, 1998). However, the unresponsiveness of PBMCs to the autologous cancer cells observed in this study does not seem to be caused by dysfunction of the effector cells in most patients. Expression of MUC1 epitopes as well as CC49-defined carbohydrate determinants on cancer cells was significantly reduced in patients who did not show the killing of autologous tumour cells. In these patients, the unresponsiveness of CTLs could be ascribed to the reduced MUC1 epitope expression on the autologous tumour cells. The PBMCs prepared from a cancer patient, which had exhibited no killing activity in initial autologous tumour cell lysis, were shown to exert significant cytotoxic activity after the expression of MUC1 mucin was recovered on the autologous cancer cells by transfecting MUC1 cDNA. This again confirmed that immune tolerance was not the major hindrance to the development of an effective cytotoxic reaction against the autologous tumour cells, and indicated that the loss of the MUC1 expression and/or antigenic modulation was the major mechanism by which autologous tumour cells escape from host immune effector cells.

MUC1 mucin is thought to be an important candidate for the target molecule in immunotherapy against cancer. Several cancer vaccines have been proposed for induction or augmentation of anti-tumour immunity, including MUC1 molecules covalently linked to the yeast cell-wall polysaccharide, synthetic peptide encapsulated with monophosphoric lipid A adjuvant, or recombinant vaccinia viruses expressing a modified MUC1 gene (Akagi et al, 1997; Karanikas et al, 1997; Samuel et al, 1998). The escape mechanism of the cancer cells due to the modulation of MUC1 antigenicity observed in this study, however, should be taken into consideration, when targeting the MUC1 mucin for immunotherapy of cancer.

### ACKNOWLEDGEMENTS

Supported in part by a Grant-in-aid for the Second Term Comprehensive Ten-year Strategy for Cancer Control from the Ministry of Health and Welfare, Japan, Grants-in-aid for Scientific Research from the Ministry of Education, Science, Sports and Culture, Japan (11680648 and on priority areas 10178104), and a grant from the Princess Takamatsu Foundation for the Promotion of Cancer Research.

The authors thank Dr. Sandra J. Gendler (Mayo Clinic Scottsdale, Scottsdale, Arizona, USA) for her kind gift of MUC1 cDNA, and Dr. Hiroshi Ikeda (Laboratory of Immunogenetics, National Institute of Animal Health, Tsukuba, Japan) for his helpful advice.

### REFERENCES

Acres B, Apostolopoulos V, Balloul JM, Wreschner D, Xing PX, Ali-Hadji D, Bizouarne N, Kieny MP and McKenzie IFC (2000) MUC1-specific immune responses in human MUC1 transgenic mice immunized with various human MUC1 vaccines. *Cancer Immunol Immunother* **48**: 588–594

Agrawal B, Reddish MA and Longenecker BM (1996) In vitro induction of MUC-1 peptide-specific type 1 T lymphocyte and cytotoxic T lymphocyte responses from healthy multiparous donors. *J Immunol* **157**: 2089–2095

Akagi J, Hodge JW, McLaughlin JP, Gritz L, Mazzara G, Kufe D, Schlom J and Kantor JA (1997) Therapeutic antitumor response after immunization with an admixture of recombinant vaccinia viruses expressing a modified MUC1 gene and the murine T-cell costimulatory molecule B7. *J Immunother* **20**: 38–47

Bara J, Imberty A, Perez S, Imai K, Yachi A and Oriol R (1993) A fucose residue can mask the MUC-1 epitopes in normal and cancerous gastric mucosae. *Int J Cancer* **54**: 607–613

Barnd DL, Lan MS, Metzgar RS and Finn OJ (1989) Specific, major histocompatibility complex-unrestricted recognition of tumor-associated mucins by human cytotoxic T cells. *Proc Natl Acad Sci USA* **86**: 7159–7163

Blockzjil A, Nilsson K, Nilsson O (1998) Epitope characterization of MUC1 antibodies. *Tumor Biol* **19** (Suppl 1): 46–56

Böhm CM, Mulder MC, Zennadi R, Notter M, Schmitt-Gräff A, Finn OJ, Taylor-Papadimitriou J, Stein H, Clausen H, Riecken EO and Hanski C (1997) Carbohydrate recognition on MUC1-expressing targets enhances cytotoxicity of a T cell subpopulation. *Scand J Immunol* **46**: 27–34

Burchell J, Wang D and Taylor-Papadimitriou J (1984) Detection of the tumour-associated antigens recognized by the monoclonal antibodies HMFG-1 and 2 in serum from patients with breast cancer. *Int J Cancer* **34**: 763–768

Burchell J, Gendler S, Taylor-Papadimitriou J, Girling A, Lewis A, Millis R and Lamport D (1987) Development and characterization of breast cancer reactive monoclonal antibodies directed to the core protein of the human milk mucin. *Cancer Res* **47**: 5476–5482

Carmon L, El-Shami KM, Paz A, Pascolo S, Tzehoval E, Tirosh B, Koren R, Feldman M, Fridkin M, Lemonnier FA and Eisenbach L (2000) Novel breast-tumor-associated MUC1-derived peptides: Characterization in $D^{b-/-}$ x β2 microglobulin (β2m) null mice transgenic for a chimeric HLA-A2.1/$D^b$-β2 microglobulin single chain. *Int J Cancer* **85**: 391–397

Dai J, Allard WJ, Davis G and Yeung KK (1998) Effect of desialylation on binding, affinity, and specificity of 56 monoclonal antibodies against MUC1 mucin. *Tumor Biol* **19** (Suppl 1): 100–110

Devine PL, McGuckin MA, Ramm LE, Ward BG, Pee D and Long S (1993) Serum mucin antigens CASA and MSA in tumors of the breast, ovary, lung, pancreas, bladder, colon, and prostate. A blind trial with 420 patients. *Cancer* **72**: 2007–2015

Finke JH, Rayman P, Alexander J, Edinger M, Tubbs RR, Connelly R, Pontes E and Bukowski R (1990) Characterization of the cytolytic activity of $CD4^+$ and $CD8^+$ tumor-infiltrating lymphocytes in human renal cell carcinoma. *Cancer Res* **50**: 2363–2370

Gendler S, Taylor-Papadimitriou J, Duhig T, Rothbard J and Burchell J (1988) A highly immunogenic region of a human polymorphic epithelial mucin expressed by carcinomas is made up of tandem repeats. *J Biol Chem* **263**: 12820–12823

Gendler SJ, Lancaster CA, Taylor J, Duhig T, Peat N, Burchell J, Pemberton L, Lalani E and Wilson D (1990) Molecular cloning and expression of human tumor-associated polymorphic epithelial mucin. *J Biol Chem* **265**: 15286–15293

Gong JL, Chen DS, Kashiwaba M, Li YQ, Chen L, Takeuchi H, Qu H, Rowse GJ, Gendler SJ and Kufe D (1998) Reversal of tolerance to human MUC1 antigen in MUC1 transgenic mice immunized with fusions of dendritic and carcinoma cells. *Proc Natl Acad Sci USA* **95**: 6279–6283

© 2001 Cancer Research Campaign

Hakomori S (1996) Tumor malignancy defined by aberrant glycosylation and sphingo (glyco)lipid metabolism. *Cancer Res* **56**: 5309–5318
Hanisch FG, Uhlenbruck G, Peter Katalinic J, Egge H, Dabrowski J and Dabrowski U (1989) Structures of neutral O-linked polylactosaminoglycans on human skim milk mucins. A novel type of lineraly extended poly-N-acetyllactosamine backbones with Gal β(1–4)GlcNAc β(1–6) repeating units. *J Biol Chem* **264**: 872–883
Ho SB, Shekels LL, Toribara NW, Kim YS, Lyftogt C, Cherwitz DL and Niehans GA (1995) Mucin gene expression in normal, preneoplastic, and neoplastic human gastric epithelium. *Cancer Res* **55**: 2681–2690
Jerome KR, Domenech N and Finn OJ (1993) Tumor-specific cytotoxic T cell clones from patients with breast and pancreatic adenocarcinoma recognize EBV-immortalized B cells transfected with polymorphic epithelial mucin complementary DNA. *J Immunol* **151**: 1654–1662
Jerome KR, Kirk AD, Pecher G, Ferguson WW and Finn OJ (1997) A survivor of breast cancer with immunity to MUC-1 mucin, and lactational mastitis. *Cancer Immunol Immunother* **43**: 355–360
Karanikas V, Hwang LA, Pearson J, Ong CS, Apostolopoulos V, Vaughan H, Xing PX, Jamieson G, Pietersz G, Tait B, Broadbent R, Thynne G and McKenzie IFC (1997) Antibody and T cell responses of patients with adenocarcinoma immunized with mannan-MUC1 fusion protein. *J Clin Invest* **100**: 2783–2792
Kontani K, Taguchi O, Narita T, Hiraiwa N, Sawai S, Hanaoka J, Ichinose M, Tezuka N, Inoue S, Fujino S and Kannagi R (2000) Autologous dendritic cells or cells expressing both B7–1 and MUC1 can rescue tumor-specific cytotoxic T lymphocytes from MUC1-mediated apoptotic cell death. *J Leukocyte Biol* **68**: 225–232
Kotera Y, Fontenot JD, Pecher G, Metzgar RS and Finn OJ (1994) Humoral immunity against a tandem repeat epitope of human mucin MUC-1 in sera from breast, pancreatic, and colon cancer patients. *Cancer Res* **54**: 2856–2860
Lan MS, Hollingsworth MA and Metzgar RS (1990) Polypeptide core of a human pancreatic tumor mucin antigen. *Cancer Res* **50**: 2997–3001
Lloyd KO, Burchell J, Kudryashov V, Yin BWT and Taylor-Papadimitriou J (1996) Comparison of *O*-linked carbohydrate chains in MUC-1 mucin from normal breast epithelial cell lines and breast carcinoma cell lines – Demonstration of simpler and fewer glycan chains in tumor cells. *J Biol Chem* **271**: 33325–33334
Magarian-Blander J, Hughey RP, Kinlough C, Poland PA and Finn OJ (1996) Differential expression of MUC1 on transfected cell lines influences its recognition by MUC1 specific T cells. *Glycoconjugate J* **13**: 749–756
Magarian-Blander J, Ciborowski P, Hsia S, Watkins SC and Finn OJ (1998) Intercellular and intracellular events following the MHC-unrestricted TCR recognition of a tumor-specific peptide epitope on the epithelial antigen MUC1. *J Immunol* **160**: 3111–3120
Ogata S, Uehara H, Chen A and Itzkowitz SH (1992) Mucin gene expression in colonic tissues and cell lines. *Cancer Res* **52**: 5971–5978
Pecher G and Finn OJ (1996) Induction of cellular immunity in chimpanzees to human tumor-associated antigen mucin by vaccination with MUC-1 cDNA-transfected Epstein-Barr virus immortalized autologous B cells. *Proc Natl Acad Sci USA* **93**: 1699–1704
Potter H, Weir L and Leder P (1984) Enhancer-dependent expression of human κ immunoglobulin genes introduced into mouse pre-B lymphocytes by electroporation. *Proc Natl Acad Sci USA* **81**: 7161–7165
Price MR, Rye PD, Petrakou E, Murray A, Brady K, Imai S, Haga S, Kiyozuka Y, Schol D, Meulenbroek MF, Snijdewint FG, von Mensdorff-Pouilly S, Verstraeten RA, Kenemans P, Blockzjil A, Nilsson O, Reddish M, Suresh MR, Koganty RR, Fortier S, Baronic L, Berg A, Longenecker MB and Hilgers J (1998) Summary report on the ISOBM TD-4 Workshop: Analysis of 56 monoclonal antibodies against the MUC1 mucin. San Diego, Calif., November 17–23, 1996. *Tumor Biol* **19** (Suppl 1): 1–20
Rowse GJ, Tempero RM, VanLith ML, Hollingsworth MA and Gendler SJ (1998) Tolerance and immunity to MUC1 in a human MUC1 transgenic murine model. *Cancer Res* **58**: 315–321
Samuel J, Budzynski WA, Reddish MA, Ding L, Zimmermann GL, Krantz MJ, Koganty RR and Longenecker BM (1998) Immunogenicity and antitumor activity of a liposomal MUC1 peptide-based vaccine. *Int J Cancer* **75**: 295–302
Siddiqui J, Abe M, Hayes D, Shani E, Yunis E and Kufe D (1988) Isolation and sequencing of a cDNA coding for the human DF3 breast carcinoma-associated antigen. *Proc Natl Acad Sci USA* **85**: 2320–2323
Smyth MJ and Ortaldo JR (1993) Mechanisms of cytotoxicity used by human peripheral blood $CD4^+$ and $CD8^+$ T cell subsets. The role of granule exocytosis. *J Immunol* **151**: 740–747
Swallow DM, Gendler S, Griffiths B, Corney G, Taylor-Papadimitrious J and Bramwell ME (1987) The human tumour-associated epithelial mucins are coded by an expressed hypervariable gene locus PUM. *Nature* **328**: 82–84
Takada A, Ohmori K, Yoneda T, Tsuyuoka K, Hasegawa A, Kiso M and Kannagi R (1993) Contribution of carbohydrate antigens sialyl Lewis A and sialyl Lewis X to adhesion of human cancer cells to vascular endothelium. *Cancer Res* **53**: 354–361
Taylor-Papadimitriou J and Finn OJ (1997) Biology, biochemistry and immunology of carcinoma-associated mucins. *Immunol Today* **18**: 105–107
von Mensdorff-Pouilly S, Verstraeten AA, Kenemans P, Snijdewint FGM, Kok A, Van Kamp GJ, Paul MA, Van Diest PJ, Meijer S and Hilgers J (2000) Survival in early breast cancer patients is favorably influenced by a natural humoral immune response to polymorphic epithelial mucin. *J Clin Oncol* **18**: 574–583
Willsher PC, Xing P-X, Clark CP, Ho DWM and McKenzie IFC (1993) Mucin 1 antigens in the serum and bronchial lavage fluid of patients with lung cancer. *Cancer* **72**: 2936–2942
Wright SE, Kilinski G, Talib G, Lowe KE, Burnside JS, Wu JY, Dolby N, Dombrowski KE, Lebkowski JS and Philip R (2000) Cytotoxic T lymphocytes from humans with adenocarcinomas stimulated by native MUC1 mucin and a mucin peptide mutated at a glycosylation site. *J Immunother* **23**: 2–10

 *© 2001 Cancer Research Campaign*
